# Longer Duration of Drug Elimination and Withdrawal Symptoms in Overweight Patients Undergoing Detoxification for Benzodiazepine Dependence

**DOI:** 10.31083/AP51895

**Published:** 2026-08-20

**Authors:** Anna Basińska-Szafrańska

**Affiliations:** ^1^Department of Prevention and Treatment of Addictions, Institute of Psychiatry and Neurology in Warsaw, 02-957 Warsaw, Poland

**Keywords:** benzodiazepine detoxification, benzodiazepine withdrawal, body mass index, SAER, serum concentration tracking, valproate

## Abstract

**Background:**

In patients undergoing detoxification for benzodiazepine (BZD) dependence, withdrawal symptoms frequently persist into the low-concentration phase following drug discontinuation. Prolonged drug elimination may extend the duration of required medical supervision. Because lipophilic BZDs can accumulate in adipose tissue and be released gradually during detoxification, overweight patients may experience delayed elimination. This study quantitatively examined whether BZD elimination and the duration of withdrawal symptoms are prolonged in overweight patients.

**Methods:**

From 508 records of concentration-monitored detoxification, a retrospective sample of 290 inpatients matched for diet and physical activity was selected. All patients underwent detoxification according to the SAER protocol (Satiation, Anti-accumulation paradigm, Elimination, and Readaptation). Following (S) satiation with diazepam, further unnecessary (A) accumulation was prevented through aggressive day-by-day dose reductions guided by serum concentration monitoring. Subsequent tapering, leading to effective (E) elimination, was adjusted according to the evolving intensity of withdrawal symptoms, characterized by episodes of peak severity (withdrawal crises) interspersed with periods of relative relief. Completion of (R) readaptation was defined by the cessation of withdrawal symptoms after elimination. The duration of effective elimination, from peak serum concentration to undetectable levels, with particular attention to the post-discontinuation phase, was recorded. Concomitant medications known to influence BZD elimination were identified. Elimination parameters and the timing of clinically significant withdrawal crises were analyzed in relation to patients' body mass index (BMI).

**Results:**

Elimination duration was positively correlated with BMI (ρ = 0.39, *p* < 0.001). The timing of the strongest and/or the final crises also showed weak but significant positive correlations with BMI in the overall sample (ρ = 0.18 and 0.15, *p* < 0.01 and < 0.05 cutoff, respectively), and within the identified co-medication subgroups, including the valproate, carbamazepine, and no-modifier groups (ρ = 0.19–0.26, all *p* < 0.05). These associations accounted for a small proportion of the variance in overall time to withdrawal crisis (R^2^ = 0.02–0.07). Nevertheless, they translated into an additional 8–11 days of necessary monitored care among obese patients. Elimination duration was further prolonged in patients receiving valproate. Extended elimination was not associated with reduced withdrawal symptom severity.

**Conclusions:**

Among patients undergoing detoxification for BZD dependence, higher BMI was associated with prolonged drug elimination and a longer duration of recurring withdrawal crises, without attenuation of symptom severity. Although the observed associations were modest and potentially influenced by confounding factors, they suggest that overweight patients may require extended monitoring during detoxification. Prospective studies are needed to confirm these findings. To reduce the risk of delayed withdrawal crises and maintain treatment within reasonable time frames, minimizing excessive BZD accumulation through laboratory-guided dose adjustment is recommended.

## Main Points

1. During detoxification treatment from benzodiazepines (BZDs), drug elimination from serum is longer in overweight patients.

2. In overweight patients with prolonged elimination of BZDs, the stage of recurring withdrawal symptoms (crises) is extended.

3. With laboratory-blind treatment, overweight patients are particularly exposed to surprising withdrawal crises after treatment conclusion.

4. Increased latency of withdrawal symptoms can be prevented by laboratory-driven action against excessive BZD accumulation (e.g. Satiation, Anti-accumulation paradigm, Elimination, Readaptation (SAER) in the present study).

## 1. Introduction

Benzodiazepines (BZDs), and similarly acting non-benzodiazepine hypnotics (Z-drugs) are consistently ranked among the top misused prescription medicines worldwide. After chronic use, 58% to 100% of users develop withdrawal symptoms at discontinuation [1]. These discourage users from further cessation attempts [2], hence exposing them to harms from deepening dependence [3]. In particular, the incidence of fatal complications due to co-administering BZD/Z with opioid analgesics has steadily grown [4]. Despite this threat, in the USA, BZDs are prescribed in >10% of the population every year, involving 7.5% of visits in primary-care and 30% visits in psychiatry-clinic settings [5]. In Europe (UK), despite sustained initiatives against BZD prescribing, the problem of long-term BZD or Z administration continues in about 1.8% or 2.3% of the general population, respectively (UK), while in some specific groups of patients, that frequency increases [6]. Across Europe, BZDs are particularly abused among the elderly [7]. In Asia, a similar trend has been observed following a recent rapid increase. The current 3.5% prevalence of long-term BZD/Z users steadily increases, especially in the young-adult population (by 9.43% yearly), but still typically remains the highest (approx. 7%) in elderly patients (Hong Kong data, [8]).

The growing need for treatment of BZD dependence has resulted in a multitude of detoxification approaches. Most of them, however, do not consider the significant inter-individual variability in BZD metabolism rate, which has led to treatment failures [9]. This situation initiated a research line on detoxification assisted by serum-BZD-concentration tracking [10,11].

The research revealed that during detoxification, successive withdrawal crises (peaks of withdrawal symptoms) may recur throughout the entire BZD elimination stage. Moreover, the symptoms often culminate in the residual concentration phase, long after drug discontinuation [10]. The resulting recommendation, to maintain medical alert until elimination (and not only drug discontinuation) is completed, is often not adhered to. The reason is that some factors, such as age, extend this process. In order to prevent surprising delayed crises occurring after the assumed treatment conclusion, the influence of possible factors should be known.

Some of the crises depend on how detoxification is carried out. Long-acting BZDs (typically diazepam), routinely introduced prior to detoxification as substitutes replacing formerly used shorter-acting drugs, are usually eliminated within 2–3 weeks after the last dose, but in some cases after 3 months or more [10]. Moreover, if such procedures do not include measures to prevent excessive accumulation of the long-acting substitute (they typically do not), drug elimination and the associated period of recurrent withdrawal crises can be much longer [11].

Some adjunct drugs administered during detoxification [12,13,14] influence the BZD elimination rate. Carbamazepine, long recognised as relieving the withdrawal symptoms [15], is a metabolism inducer. Valproate, sometimes used for the detoxification process [16,17], increases the free fraction of diazepam nearly 2-fold by displacing it from plasma-protein binding and increases diazepam plasma clearance [18]. On the other hand, valproate inhibits hepatic metabolism of diazepam [18,19], influencing cytochrome P450 family 2, subfamily C, member 9 (CYP2C9) [20,21], CYP3A4, and CYP2C19 [21]. Its own clearance may depend on the dose and the patient’s sex or weight [22]. Hence, the final impact of valproate on diazepam elimination depends on the balance of all these factors. This diversity might have contributed to the divergent research results on valproate thus far (reviewed in [19]). In BZD-dependent patients using valproate as adjunct medication, two studies using the same substitute BZD and the same detoxification method showed divergences in some secondary results [9,10]. *Ex post* analyses revealed uneven group matching in terms of body mass index (BMI), which inspired the idea of the present study.

Adiposity may be an underestimated [23] but relevant factor in detoxification from BZDs, as most BZDs are lipophilic drugs. This matters especially when a long-acting substitute is used, such as diazepam or clorazepate. Due to redistribution, a greater volume of an individual’s adipose-tissue compartment may result in lower BZD concentrations in serum, or in higher doses needed to achieve the required effect. During detoxification, a release of BZD deposits likely extends the elimination process [24,25,26]. Moreover, in higher-BMI patients, the activity of CYP3A4 and CYP2C9 enzymes is decreased, although weight loss may increase the activity of CYP3A4, the main diazepam-metabolising enzyme [27].

The hypothesis tested in this study was that in BZD-dependent patients with adiposity, their higher adipose-tissue content (represented here by BMI as a measurable proxy) is associated not only with prolonged elimination of BZD after chronic use, but also with the delayed latency of recurring withdrawal crises.

This retrospective, open, semi-naturalistic study was based on available records of treatment using serum-BZD-concentration tracking. It was conducted under the standardized conditions of a closed detoxification ward, with procedures designed to prevent excessive accumulation [28]. The concomitant medication, including elimination modifiers, was taken into account.

## 2. Materials and Methods

### 2.1 Patients

A total of 508 completed detoxification records from inpatients meeting the International Classification of Diseases, 10th Revision (ICD-10) criteria for BZD dependence [29] were analyzed retrospectively. The treatment procedure was performed with the patients’ informed consent. The records, collected in order of admissions, in uniform conditions of closed-ward daily routine, documented the detoxification courses of typical patients in a detoxification ward, including those with coexisting somatic and psychiatric conditions. Typically, patients with BZD dependence often reported a history of insomnia, anxiety or mood disorders, or alcohol abuse. However, prior to their scheduled admission, a stable mental and somatic state was required. Therefore, the patients maintained their basic treatment (**Supplementary Table 1**), but those using medication that significantly influenced BZD metabolism were noted (then analyzed separately). Patients entering the treatment could not be in an active phase of dependence on drugs other than BZDs, and stable abstinence (no withdrawal symptoms) from substances other than BZDs, confirmed also by breathalyzer and urine drug-screening tests, was required at admission.

**Fig. 1.**
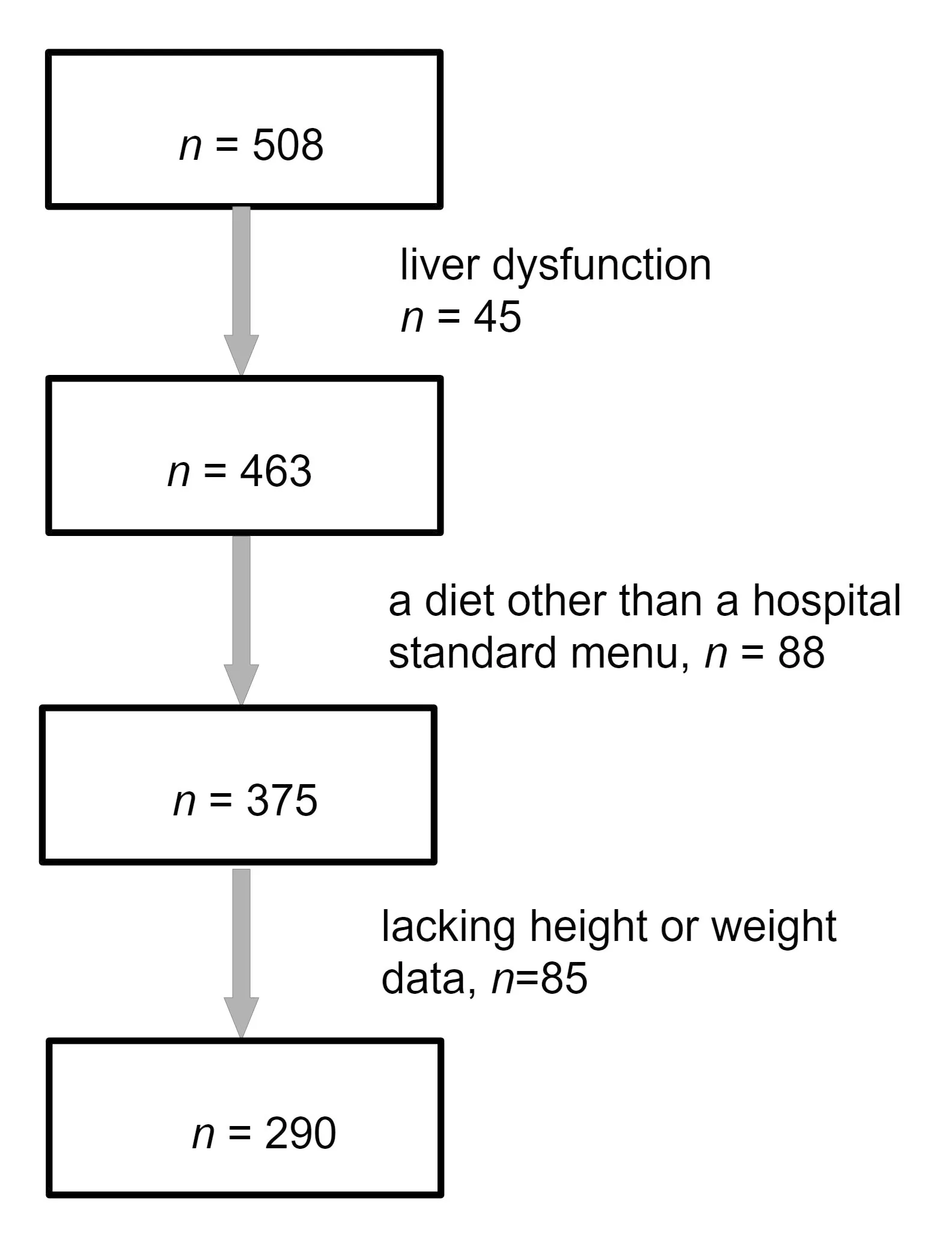
**The criteria for selecting the study records**.

From this sample, in addition to the patients with evidence of liver dysfunction, patients on special diets were excluded in order to eliminate the uneven influence of food on drug effects [30,31,32,33]. Inpatient conditions practically eliminated the influence of differences in motor activity [33]. Further exclusion of records with lacking weight or height data reduced the investigated sample to 290 inpatients (Fig. 1).

### 2.2 Procedure

The records involved the 4-stage “Satiation, Anti-accumulation paradigm, Elimination, Readaptation (SAER)” detoxification procedure as the first regular procedure assisted by serum BZD tracking (see [28] for details; see **Supplementary Fig. 1**). The procedure was accepted by the institution’s Bioethical Committee and routinely implemented in its detoxification ward*.*

#### 2.2.1 Substitution (S)

As a long-acting substitute BZD, diazepam was chosen for its good traceability (including active metabolites) in the available laboratory test. Substitution was a one-day process and started at least 8 h after the last previous-drug dose. The initial (loading) diazepam dose satiating dose (d_SAT_) was completed using a titration procedure with 5–20 mg diazepam doses at interdose intervals of 1.5–2 h up to the patient’s reported and observed satiation state [34]. Satiation was assessed based on the patient’s self-reported “normal” well-being, with the physician verifying the patient’s report by referring it to the clinical presentation (to exclude euphoria, oversedation, or dissimulation). After unanimous assessment, that state was then quantified with both the Clinical Institute Withdrawal Assessment Scale - Benzodiazepines (CIWA-B) questionnaire [35] and the serum BZD concentration level satiation concentration (C_SAT_), serving as the individual clinical-state and concentration baselines. In order to obtain a CIWA-B score, the patient assessed the current severity (last 24 h, 0–4 scale) of 17 symptoms (plus 3 symptoms assessed by the doctor). For serum BZD measurements, taken after confirming a clinical satiation state in the morning prior to the first meal, the typically available assay was used serum benzodiazepine (SBENZ) immunoassay/COBAS Integra 400 plus analyzer, Roche Diagnostics; limit of detection (LoD): 3 ng/mL, precision: 5.5% coefficient of variation (CV), accuracy: 100% for SBENZ-negative results, 100% for SBENZ-positive results for gas chromatography/mass spectrometry (GC/MS)-positive samples, and 10.8% for SBENZ-positive results for GC/MS-negative samples [36].

#### 2.2.2 Anti-Accumulation Paradigm (A)

After the C_SAT_ was achieved and measured, further (unneeded) accumulation was actively minimised. The doses were reduced daily, beginning with the subtraction of approximately 30% of the loading dose. The subsequent daily dose-reducing steps were approximated from the daily concentration feedback until accumulation stopped (a few days). The serum samples were collected every morning, approximately 8 h after the previous night’s dose (in a concentration trough), before the first meal. The peak accumulation (C_ACC_) and the day of its occurrence (D_ACC_), counted from the start of treatment, were noted.

#### 2.2.3 Elimination (E)

The further dose reduction started the effective elimination, hence tapering became slower and flexibly adjusted to the reported and observed patient’s clinical state. Continuous medical assistance to alleviate withdrawal symptoms, especially during their culminations (crises), was sustained after drug discontinuation, to the end of the elimination process. Any marked intensity or change in the patient’s condition was quantified using the CIWA-B score involving the past 24 hours. If needed, accessory medication was allowed (**Supplementary Table 2**). At this stage, serum checks (every 3–7 days) served to track the elimination course. Decline of the serum BZD below the trackable level (<3 ng/mL) was tentatively adopted as the end of the stage.

#### 2.2.4 Readaptation (R)

This post-elimination stage was intended to assist patients with their continuing withdrawal symptoms up to the patients’ readaptation to abstinence (approaching the CIWA-B baseline). Up to this point, the supportive medication adjusted for currently dominating ailments was gradually discontinued. Blood samples were taken only at random, as abstinence controls.

### 2.3 Data Elaboration

For quantification of the flexible detoxification course, some essential variables were defined:

d_SAT_, C_SAT_ – initial (loading) diazepam dose and the corresponding satiating serum BZD concentration (the concentration baseline);

D_ACC_, C_ACC_ – the day (counted from the beginning of treatment) and the level of maximal serum BZD accumulation;

D_W_ – the withdrawal day (next day after the last diazepam dose, counted from the beginning of treatment);

D_E_ – the elimination day (the first day of BZD concentration descent below the detection level);

D_ACC_ to D_E_ and D_W _to D_E_ – time (days) to elimination completion D_E_, elapsed from the start of effective elimination (D_ACC_) and from drug discontinuation (D_W_), respectively;

D_MAX_ and D_LAST_ – the day of the maximal and the last individual withdrawal crisis, respectively, indicated by relative extrema (the worst days) in the individual CIWA-B-score series. In case of uncertainty (e.g., at the plateau of the score), the patients and doctor determined the worst day retrospectively. D_MAX_ and D_LAST_ could be the same.

Serum-BZD levels and CIWA-B scores were assessed compared to individual baselines.

Treatment-related data were referred to patient-related data: age, height, weight, and BMI, used as continuous variables.

Finally, either patient data or treatment (elimination) data were referred to the timing of the strongest (D_MAX_) and the last (D_LAST_) withdrawal crises.

In case of revealing a subgroup of patients receiving, as their basic treatment, a medication modifying BZD elimination rate, their patient- and treatment-related data were compared with those in the main group of patients, in order to check inter-group matching for purposes of inter-group comparisons.

### 2.4 Statistical Analysis

Due to the asymmetrical distribution of the data, nonparametric statistics were applied. Results for the groups were represented by medians and interquartile ranges. For the multi-group comparisons, significant (*p* < 0.05) differences were detected using Kruskal‒Wallis rank analysis and followed by Mann‒Whitney tests within each pair of groups. Correlations between patient- and treatment-related data were examined using the Spearman rank correlation test. The tests were performed using Statistica software version 13.3 (StatSoft Polska Sp. z o.o., Krakow, Poland) [37].

## 3. Results

Preliminary analysis of the extracted data, with respect to an elimination modifier used, revealed 113 patients receiving valproate (the VAL group) and 119 patients receiving carbamazepine (the CBZ group), which, in addition to the remaining 58 patients (the NONE group), formed three medication groups. These groups were well matched with respect to the relevant patient-related data, since Kruskal‒Wallis analyses revealed no significant differences between the groups in age, duration of addiction, BZD doses, and the main patient-related factors studied: weight, height, and BMI (Table 1).

**Table 1.** **Characteristics of the study sample (available data)**.

Group	NONE (*n* = 58)	VAL (*n* = 113)	CBZ (*n* = 119)	H	*p*
Sex (% of males)	46	52	42		
Age	52.0 [35.5–61] 49.4 (15.2)	48.0 [35.5–58.0] 47.8 (14.5)	48.0 [40.0–58.0] 49.8 (14.4)	2.160	ns
Years of addiction	7.0 [4.0–10.0] 8.3 (6.1)	8.0 [4.0–13.0] 10.1 (8.1)	9.5 [4.0–15.0] 10.8 (8.1)	1.159	ns
BZD dose (the initial dose of diazepam needed) [mg]	25.0 [5.0–40.0] 40.4 (29.1)	30.0 [15.0–50.0] 33.0 (22.7)	27.0 [15.0–50.0] 34.8 (27.2)	1.410	ns
Weight [kg]	72.5 [59.0–83.0] 74.1 (18.9)	73.3 [64.3–89.0] 77.9 (21.2)	75.3 [63.1–87.5] 76.0 (17.1)	1.969	ns
Height [m]	1.68 [1.64–1.73] 1.69 (0.08)	1.70 [1.64–1.76] 1.70 (0.09)	1.69 [1.62–1.78] 1.70 (0.10)	1.048	ns
BMI	24.6 [21.5–30.0] 25.6 (5.1)	25.8 [22.5–30.0] 26.7 (5.8)	25.8 [23.2–28.7] 26.1 (5.0)	1.309	ns

Median values and interquartile ranges (square brackets) within the no-modifier (NONE), valproate (VAL), and carbamazepine (CBZ) groups are presented, with no significant differences between the groups. Means and standard deviations (lower lines) are included for informative purposes only. BZD, benzodiazepine; BMI, body mass index; ns, statistically non-significant difference.

With respect to the initial treatment-related data (a start point to the effective elimination process), comparing the groups revealed no significant differences with respect to d_SAT_, C_SAT_, and D_ACC_ median values. However, the C_ACC_ level was significantly higher in the carbamazepine group than in the “NONE” group (725 ng/mL [interquartile 394–1282] vs. 382 ng/mL [161–1148], Z = 1.99, *p* = 0.046), revealing that the carbamazepine group was not fully matched in terms of initial conditions for the elimination process.

The elimination-related data compared among the 3 comedication groups confirmed the expected differences in the entire duration of effective elimination (H = 11.107, *p* = 0.004) and in the phase after drug discontinuation (H = 11.806, *p* = 0.003). Paired analyses confirmed a longer effective elimination duration (D_ACC _to D_E_) in the valproate group (median 29 days, interquartile [22–42]) than in the “NONE” (21 [18–36], Z = 2.56, *p* = 0.010) and carbamazepine groups (26 [21–33], Z = 2.47, *p* = 0.014), and a longer elimination phase after drug discontinuation (D_W _to D_E_) in the valproate group (23 [15–36]) than in the no-modifier (18 [10–30], Z = 1.97, *p* = 0.048) or carbamazepine groups (18 [13–24], Z = 3.08, *p* = 0.002). Contrary to expectations, the total elimination duration in the carbamazepine (metabolism-inducer) group was not the shortest. This was explainable, at least in part, by the abovementioned higher pre-elimination level (C_ACC_) in this group. Finally, although the data remained acceptable for intra-group analyses, the carbamazepine group was excluded from intergroup comparisons.

For expository purposes, the time sequence of the consecutive treatment milestones is presented in **Supplementary Table 3**.

After the preliminary assessment of the collected sample, the analyses focused on the main topic of the study. First, it was confirmed that the known relationship between longer elimination and later occurrence of crises [10] was replicated in the overall study sample and across groups (Table 2).

**Table 2.** **The correlations within the study sample between the elimination results and the day of the last withdrawal crisis, D_LAST _(A), or the day of the maximal (most intense) one, D_MAX _(B)**.

**(A)**				
	D_LAST_			
	TOTAL n = 290 ρ, *p*	NONE n = 58 ρ, *p*	VAL n = 113 ρ, *p*	CBZ n = 119 ρ, *p*
The final day of elimination, D_E_	+0.53 <0.001	+0.58 <0.001	+0.58 <0.001	+0.48 <0.001
The duration of the effective elimination, D_ACC_ to D_E_	+0.51 <0.001	+0.53 <0.001	+0.57 <0.001	+0.47 <0.001
Elimination after drug discontinuation, D_W_ to D_E_	+0.46 <0.001	+0.45 =0.001	+0.58 <0.001	+0.36 <0.001
**(B)**				
	D_MAX_			
	TOTAL n = 290 ρ, *p*	NONE n = 58 ρ, *p*	VAL n = 113 ρ, *p*	CBZ n = 119 ρ, *p*
The final day of elimination, D_E_	+0.27 <0.001	+0.28 <0.05	+0.31 =0.001	+0.28 <0.01
The duration of the effective elimination, D_ACC_ to D_E_	+0.27 <0.001	+0.27 <0.05	+0.33 <0.001	+0.28 <0.01
Elimination after drug discontinuation, D_W_ to D_E_	+0.25 <0.001	+0.27 <0.05	+0.31 =0.001	+0.29 <0.01

As confirmed either for the entire study sample or for the 3 comedication subgroups, these correlations underlie the respective secondary relationships between patient-related elimination cofactors (age, BMI) and latencies of the crises (Tables 3,4,5,6). Abbreviations indicate days counted from the first day of the procedure: D_E_, day of completed elimination; D_ACC_, day of maximum BZD concentration; D_W_, day of diazepam discontinuation; D_MAX_ and D_LAST_, days of maximum and last crisis, respectively.

Next, analyses examined whether this substrate relationship, along with the expected “BMI × elimination” relationship, translated into a “BMI × timing of the crises” interaction. In the entire study sample (Table 3) and in each of the co-medication groups (Tables 4,5,6), the duration of effective elimination (D_ACC _to D_E_) significantly correlated with BMI and, to a lesser extent, with patients’ weight but not height. Correlations between elimination duration and BMI were stronger in the period after the drug discontinuation (D_W_ to D_E_). In the entire sample and in each comedication group, the correlations of the elimination data with BMI were stronger than those with age (a known cofactor [10]). For clarity, correlations between BMI and age were excluded (as nonsignificant) within any group. Therefore, a function of BMI and age, the body fat percent BF%, was introduced, as related to the body fat content more specifically (the last column in Tables 3,4,5,6).

**Table 3.** **The results for the overall sample**.

TOTAL *n* = 290	Age	Weight	Height	BMI	BF%
The final day of elimination, D_E_	+0.23, <0.001	+0.32, <0.001	ns	+0.41, <0.001	+0.37, <0.001
The duration of the effective elimination, D_ACC_ to D_E_	+0.24, <0.001	+0.27, <0.001		+0.39, <0.001	+0.37, <0.001
Elimination after drug discontinuation, D_W_ to D_E_	+0.33, <0.001	+0.24, <0.001		+0.40, <0.001	+0.44, <0.001
The day of the strongest crisis, D_MAX_	+0.13, <0.05	+0.15, <0.05		+0.18, <0.01	+0.15, <0.05
The day of the last crisis, D_LAST_	+0.28, <0.001	ns		+0.15, <0.05	+0.28, <0.001
The age × BMI correlation was nonsignificant					

Correlations (ρ, *p*) between patient-related data and pharmacokinetic (PK)-related (elimination) or pharmacodynamic (PD)-related (timing of crises) data. The last column includes similar tests using BF% as an alternative to BMI. Abbreviations indicate days counted from the first day of the procedure: BF%, body fat percent; ns, statistically non-significant difference.

**Table 4.** **The results for the “NONE” group, receiving no medication influencing the BZD elimination rate**.

NONE group *n* = 58	Age	Weight	Height	BMI	BF%
The final day of elimination, D_E_	ns	+0.30, <0.05	ns	+0.42, <0.01	+0.31, <0.05
The duration of the effective elimination, D_ACC_ to D_E_	ns	ns		+0.38, <0.01	+0.37, <0.01
Elimination after drug discontinuation, D_W_ to D_E_	+0.33, <0.05	ns		+0.41, <0.01	+0.42, <0.01
The day of the strongest crisis, D_MAX_	ns	+0.32, <0.05		+0.26, <0.05	ns
The day of the last crisis, D_LAST_	ns	ns		ns	ns
The age × BMI correlation was nonsignificant					

Correlations (ρ, *p*) between patient-related data and PK-related (elimination) or PD-related (timing of crises) data. Due to the smaller group size, *p* values are higher. The last column includes similar tests using BF% as an alternative to BMI. ns, statistically non-significant difference.

**Table 5.** **The results for the valproate (VAL) group**.

VAL group *n* = 113	Age	Weight	Height	BMI	BF%
The final day of elimination, D_E_	+0.28, <0.01	+0.34, <0.01	ns	+0.38, <0.001	+0.37, <0.001
The duration of the effective elimination, D_ACC_ to D_E_	+0.26, <0.01	+0.29, <0.01		+0.40, <0.001	+0.39, <0.001
Elimination after drug discontinuation, D_W_ to D_E_	+0.36, <0.001	+0.28, <0.05		+0.41, <0.001	+0.43, <0.001
The day of the strongest crisis, D_MAX_	ns	ns		ns	ns
The day of the last crisis, D_LAST_	+0.31, <0.01	ns		+0.19, <0.05	+0.24, <0.05
The age × BMI correlation was nonsignificant					

Correlations (ρ, *p*) between patient-related data and PK-related (elimination) or PD-related (timing of crises) data. The last column includes similar tests using BF% as an alternative to BMI. ns, statistically non-significant difference.

**Table 6.** **The results for the carbamazepine (CBZ) group**.

CBZ group *n* = 119	Age	Weight	Height	BMI	BF%
The final day of elimination, D_E_	+0.23, =0.01	+0.30, <0.01	ns	+0.42, <0.001	+0.46, <0.001
The duration of the effective Elimination, D_ACC_ to D_E_	+0.23, < 0.05	+0.24, <0.05		+0.35, <0.001	+0.39, <0.001
Elimination after drug discontinuation, D_W_ to D_E_	+0.32, <0.001	+0.22, <0.05		+0.39, <0.001	+0.49, <0.001
The day of the strongest crisis, D_MAX_	ns	ns		+0.21, <0.05	+0.22, <0.05
The day of the last crisis, D_LAST_	+0.29, <0.05	ns		ns	+0.36, <0.001
The age × BMI correlation was nonsignificant					

Correlations (ρ, *p*) between patient-related data and PK-related (elimination) or PD-related (timing of crises) data. The last column includes similar tests using BF% as an alternative to BMI. ns, statistically non-significant difference.

To exclude the influence of the initially higher C_ACC_ level in the CBZ group on the potential correlation modeling for the entire study sample, the key correlations from Table 3 were repeated for the NONE+VAL group only, as a sensitivity test (see **Supplementary Table 4A** vs. **Supplementary Table 4B**).

Consistently (Table 2), correlations between BMI and elimination data translated into (weaker) secondary correlations between BMI and timing of withdrawal crises (Tables 3,4,5,6). Although the latency of the last crisis typically correlated with age [10], the latency of the most severe crisis more often (except for the valproate group) correlated with BMI. The latencies of the crises did not significantly correlate with their intensity, measured both as absolute CIWA-B scores or relative to individual baselines.

Scatterplots presenting individual results and regression lines have been provided in **Supplementary Fig. 2**, in **Supplementary Analysis**. It was noted that in the BMI range 32+, the data points are scarce but significantly affect the regression line, skewing it in the direction opposite to the overall correlation. Considering the possibility that these values are not representative of the given BMI ranges, the same analyses were repeated after limiting the data to the BMI range of 32 (**Supplementary Fig. 3**, in **Supplementary Analysis**). In that case, the correlations become stronger and are well represented by linear regression. The relationships in the BMI 32+ range remain uncertain.

**Fig. 2.**
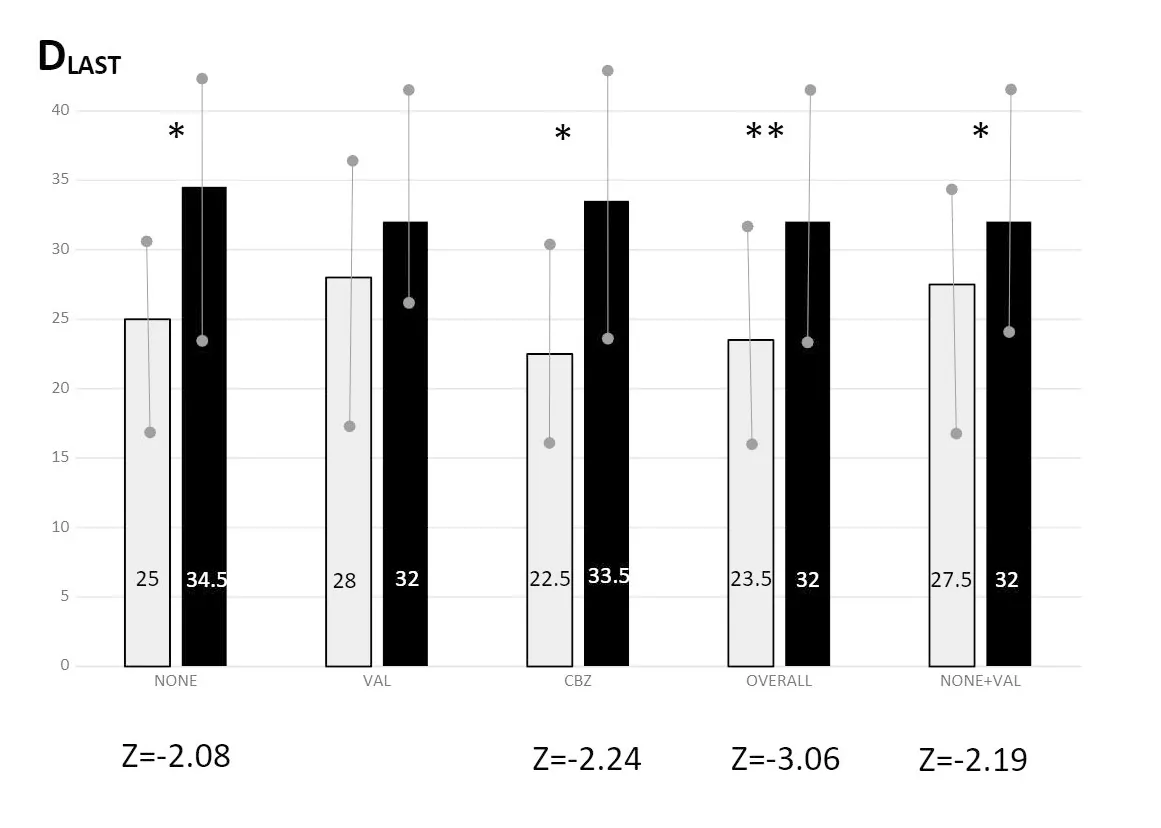
**An illustrative example of BMI-correlated differences in the median time to the occurrence of the last withdrawal crisis**. Days to the last crisis, D_LAST_, are presented for the two wider spaced (1 vs. 4) BMI quartiles. Significant differences in the median values between the lowest (up to 22.8 here) and the highest (30+) BMI ranges within each study group are indicated by asterisks (* for *p* < 0.05, ** for *p* < 0.01). Weak but significant correlations revealed in the study, except for the valproate group where elimination was prolonged, still translate into a (median) 8–11 day delay in the crisis’s occurrence for obese patients. (Blurred differences between the quartiles in the valproate group automatically weaken the result for the NON+VAL population.) For the most intensive crisis, D_MAX_, similar differences were not significant.

With respect to the timing of the crises, the distribution of the results (large scatter, weakening the correlations) and R^2^ up to 0.07 (higher for BF%) indicated that both BMI and BF% contributed only a small part of the observed variability (other factors’ influence). To assess the practical value of the results, a comparison for patients representing the extreme BMI interquartile ranges is presented for expository purposes in Fig. 2. The correlations, even though weak, still resulted in (median) 8 or 11 days longer monitored assistance in obese patients (BMI ≥30, 4th quartile) compared to patients with BMI <22.8 (1st BMI quartile). In this comparison differences for D_MAX_ weakened to a trend only (the 4th quartile includes the abovementioned controversial 32+ range).

For the overall sample, a similar comparison for the day of elimination D_E_ is 27.0 [21.0–33.0] vs. 40.5 [31.5–50] (Z = –6.10; *p* < 0.001), for elimination duration (D_ACC_ to D_E_) 22 [18.25–28] vs. 33 [27.0–46.0] days (Z = –5.51; *p* < 0.001), and for elimination after diazepam discontinuation (D_W_ to D_E_) 15 [10.25–21.0] vs. 29 [18.0–40.5] days (Z = –5.39; *p* < 0.001). An overall comparison for elimination and crises timing is provided in Table 7.

**Table 7.** **The comparison of elimination and crises-timing results between patients representing the 1st and the 4th BMI quartiles**.

	1st BMI quartile <22.8 (median, interquartiles)	4th BMI quartile ≥30 (median, interquartiles)	Z; *p*
The final day of elimination, D_E_	27.0 [21.0–33.0]	40.5 [31.5–50.0]	–6.10; <0.001
The duration of the effective elimination, D_ACC_ to D_E_	22 [18.25–28.0]	33 [27.0–46.0]	–5.51; <0.001
The duration of elimination after drug discontinuation, D_W_ to D_E_	15 [10.25–21.0]	29 [18.0–40.5]	–5.39; <0.001
The day of the strongest crisis, D_MAX_	14 [7.25–25.75]	20 [10.0–28.0]	–1.94; 0.052
The day of the last crisis, D_LAST_	23.5 [16.0–32.0]	32 [23.75–42.0]	–3.06; <0.01

The results for the overall study sample.

Consistently with no significant correlations between time to occurrence and intensity of the crises, similar comparison between median CIWA-B values revealed no significant difference between the 1st and 4th BMI quartiles in intensity of the worst crisis (CIWA-B score 41 [35.5–51] vs. 43 [35.5–50], ns) and the last one (35 [22.5–44.5] vs. 38 [22–46.5], ns).

## 4. Discussion

It was not surprising that the study first confirmed the initial assumption that adiposity affects the time to BZD elimination. Furthermore, the already known relationship between the time to elimination completion and latency of the decisive withdrawal crises [9,10,38] was confirmed in this study sample. Finally, the study supported the hypothesis that overweight patients, especially those detoxified in nonmonitored conditions, are at significant risk of unexpectedly delayed withdrawal-symptom crises. A similar problem has been described in patients with alcohol-withdrawal syndrome treated with diazepam [39].

The data were expected to carry some noise due to a multitude of possible causative factors and because data gathering was not supported by procedures of a formal clinical test. Therefore, their significance seems to be particularly relevant.

Longer elimination time correlated with higher BMI and, to a lesser extent, with greater weight of patients, although it did not show significant relationships with their height. This strongly suggested that it is adiposity, and not the overall volume of a tissue compartment, that modified the rate of BZD elimination. If a calculated BF%, determining fat content more specifically, replaced BMI as the independent variable, the correlations studied were similar, and some (related to timing of the crises) were better.

The relationship between BMI and elimination duration was significant despite some interfering medical actions that made the correlations weaker. In this flexible approach (a limitation of the study), the effective elimination duration (starting with peak accumulation, D_ACC_) depended, in part, on the pace of diazepam tapering. This, in turn, was flexibly adjusted to patients’ clinical condition, reflecting the individual readaptation process (additional “noise”). That is probably why the relationship was stronger in the less disturbed phase (after BZD discontinuation) of the elimination process. Nevertheless, it was significant despite the fact that in this study, lean patients (1st BMI quartile) discontinued diazepam on average 3 days later (counting from D_ACC_) than obese ones (4th quartile). The prolonged elimination of BZDs in overweight and obese patients may be important in many other circumstances. For example, it may be responsible for unintended driving-under-the-influence charges many days after BZD-treatment cessation, outside the addiction context.

The BMI-correlated delay of the low-concentration detoxification stage appeared to translate into a BMI-correlated delayed appearance of decisive withdrawal crises. The latter, as hypothetically secondary relationships, were weaker than the former one, but still significant. The significance coefficients varying between the groups plausibly resulted from statistical issues (different group sizes): they are typically highest in the smallest group and lowest for the entire study sample. However, these correlations were surprisingly weak, and BMI explained only a small proportion of the variance in the final time-to-crisis (up to 3%). A bit better impact (up to 7%) from BF% can be a motivation for further studies. In the valproate group, where elimination was significantly longer, the differences in the time-to-last-crisis between lean and obese patients were blurred, which may be a result to explore. The other factors (confounders in this study) are discussed in the Limitations subsection. As in some part unavoidable in semi-naturalistic research conditions, they limited but did not eliminate the practical value of the study results. In the obese patients (BMI 30+; 4th quartile) in this study sample, they still translated into a median delay in the last crisis’ appearance of 8.5 days, and even 11 days in the carbamazepine group (Fig. 2).

The warning against a delayed withdrawal crisis was not obvious, as prolonged elimination is usually expected to ease the course of detoxification. The study data did not support that expectation. Actually, the real clinical scenario during elimination depends on individual differences between the elimination slope and adaptation rate. The delayed elimination completion may have resulted either from a slow concentration decline (long and easy course) or from a delayed but steep concentration drop (late but intense crisis). The latter may occur after initial overaccumulation of a long-acting substitute, which is highly probable during a non-monitored detoxification [9,11].

However, in this study, despite the anti-accumulation paradigm used, “delayed but not weaker” crises still occurred (no correlations between latency and intensity). Hypothetically, with overweight patients, the process of releasing BZDs from adipose-tissue deposits may have replenished declining serum BZD levels. The transition between the compartments effectively prolonged the elimination of lipophilic drugs in obese patients [40,41], but there is little data about the dynamics of this process. The authors hypothesize that transfer from the peripheral compartment and elimination from the central one may not be smooth or synchronous. With more erratic release from the adipose tissue, the effective elimination rate from serum may vary, with episodes of faster elimination. Such instability, in turn, might hinder individual adaptation processes. This hypothesis should be verified under standardized conditions. Nevertheless, in some obese patients monitored in the present study, during elimination, transient periods of concentration plateaus or even incidental minor increases were observed. Even though these could be attributed to a lower precision of semi-quantitative measurements, such phenomena were not observed in lean patients. This observation is worth attention because an incidental concentration increase during detoxification usually raises concerns (often correct) about a patient’s unauthorised drug use. In obese patients, the intercompartment transition issues are considerable.

In everyday practice, adiposity may interact with other elimination cofactors, which is both risky and beneficial, opening some avenues for improvement of the process. In older or obese patients, using valproate to secure a detoxification course may waste their treatment time on an extended high-concentration phase. However, in rapidly eliminating patients, if the adaptation process cannot keep up, slowing post-withdrawal elimination may provide time for the readaptation process. In particular, valproate may be a good choice in lean patients, in whom an additional impact of the fat compartment, if any, can be irrelevant. Conversely, lean patients (and/or with hypoalbuminemia), in contrast to obese ones, may not benefit from the metabolism inducer carbamazepine, even though it is rightly recommended as an adjunct medication mitigating BZD-withdrawal symptoms [15,42].

The current results may have explained some previous inconsistent BZD-elimination data from patients receiving valproate ([11] vs. [10]). The retrospective check of the study sample [11] with respect to BMI (originally not examined) revealed significantly lower BMI in the valproate group than in the no modifier and carbamazepine groups (23 vs. 25 and 25), resulting in a blurring of the elimination-prolonging valproate effect.

The primary advantage of the study was the data coming from detoxification employing SAER, the first regular procedure actively minimizing the initial overaccumulation of a long-acting substitute (a potent confounder) and adjusting the treatment to highly diverse BZD-metabolism rate (another confounder). The anti-accumulation paradigm (A) increased the reliability of any clinical or pharmacokinetic results considering detoxification from BZDs. The procedure has been proposed as a novel framework to be used and developed in both treatment and research settings [28].

### 4.1 Limitations

One limitation of the study was its retrospective character: the data collection process, even though running under the uniform in-ward conditions, was not specifically oriented to the topic of the study. Any elements that distorted the real-time precision in determining a day of a given crisis could have specifically weakened the obtained correlations for D_MAX_ or D_LAST_.

A selection of records may have introduced selection bias, limiting the conclusions to a relatively healthier subset of the sample. Lean patients were underrepresented in the study population (see median BMI values). More importantly, high BMI ranges were insufficiently represented, thereby weakening the observed effect (in **Supplementary Table 4B**). The authors recommend ensuring adequate representation of BMI extremes in future prospective studies.

The carbamazepine group data, as not fully matched with respect to initial conditions for elimination, were excluded from inter-group comparisons, and within-group analyses should be interpreted more cautiously.

Auxiliary drugs, although selected from a limited set of medications, varied in composition depending on the current withdrawal symptom profile, and their impact was not analyzed here. Considering fixed-use medications, the effect of valproate alone on BZD elimination depends on the combined impact of several cofactors [19].

Regardless of the pharmacokinetic (PK) issues, the occurrence and intensity of the crises were related to pharmacodynamic (PD) issues, as associated with receptor dysregulation. In this study, groups were matched for duration of addiction (based on patient interview) and the tolerance developed, but explicit tests for addiction severity (proxy for PD issues) were not done.

The flexible approach, advantageous as a treatment method, affected the collected data and relations examined. A fixed-tapering schedule could reduce the impact of some confounders (symptom-dependent tapering, a choice of a day of the BZD discontinuation). However, interindividual differences (metabolism rate, dose tolerance, and tolerance of withdrawal symptoms) encourage individualization of treatment.

Despite all the limitations, the correlations regarding the last crisis, although quantitatively weakened by confounders, qualitatively remained robust in sensitivity testing. Eliminating CBZ from the overall analyses preserved the key correlations (**Supplementary Table 4**), and the exclusion of outliers (see **Supplementary Analysis**) made them stronger.

### 4.2 Closing Remarks

The flexible-study procedure, indicated above as a limitation, actually addressed phenomena that have not been taken into account for decades. In any nonmonitored detoxification after long-acting BZD substitution, the largest problem is an initial BZD overaccumulation. It fundamentally determines initial conditions for the elimination process (latency of its start, and initial concentration level) and the course of the entire further detoxification treatment, including a prolonged period of drug influence and the withdrawal crises. In this study, overaccumulation was roughly curbed, but in common practice, it is uncontrolled.

Flexible adjusting of the procedure to ongoing laboratory feedback addressed and compensated for many PK-factors recognized or unknown. In the anti-accumulation paradigm, consecutive dose-reducing steps adjusted the procedure to the individual (highly diverse) metabolism rate, determined genetically and affected by other factors. As their resultant is finally expressed by the current concentration result, the clinician’s response spontaneously compensates for their influence. Therefore, tracking serum-BZD concentration (as in SAER) allows for compensatory interventions and evidence-based dose regulation [23].

This approach, controlling for PK variability, should extend, if practical, to any treatment using BZDs. Concentration should be measured [43] and interpreted correctly. In obese patients, and not only with respect to BZDs, the drug concentration monitoring is particularly important, as the drug approval process often neglects significant differences in PK in these patients [44].

## 5. Conclusions

During detoxification of overweight patients dependent on BZDs, both elimination and the related period of recurring withdrawal crises may be prolonged. The results of this study, based on treatment records, revealed statistically robust moderate correlations between adiposity (as BMI) and prolonged BZD elimination. The contribution of the “BMI signal” to the timing of the last crisis was weak, accounting for several percent of the crisis delay variation. Despite this dominating impact of confounders, the results still translated into a median of 8–11 days of longer need for medical assistance in obese patients. To prevent premature treatment conclusion and unassisted further (delayed) crisis, recognizing the current crisis as the last should be conditional on the end of elimination. To maintain all in-ward treatment in overweight patients within reasonable time limits, minimization of excessive BZD accumulation should be under laboratory feedback. In patients treated with valproate, elimination may be prolonged without reducing crisis intensity. If needed, a temporary alternative medication should be carefully considered.

The current study highlighted a scarcely researched problem that requires thorough investigation in a prospective study. Flexible treatment procedures are necessary, but need to be refined in order to minimize the influence of confounders and to provide refined research data.

## Data Availability

The records analysed during the current study are not available publicly due to local data protection regulations but available from the corresponding author on a reasonable request.
