# Supplementary material for: Longer Duration of Drug Elimination and Withdrawal Symptoms in Overweight Patients Undergoing Detoxification for Benzodiazepine Dependence
Source: Alpha Psychiatry. 2026 Aug 20;27(4):51895. doi: 10.31083/AP51895 (PMC13540023; doi:10.31083/AP51895)
Supplement: Supplementary file 1 [file 2757-8038-27-4-51895-s1.zip › Suplementary Materials.docx]

Supplementary Table 1

The most frequent concomitant psychiatric medications (Table 1A) or medications for somatic conditions (Table 1B) accepted and continued throughout the study. In particular, the anticonvulsant agents, formerly introduced, could not be withdrawn at the detoxification onset.

Table 1A.

| Medication | Condition | Application |
| --- | --- | --- |
| valproate | bipolar mood disorder  other mood disorders  benzodiazepine dependence | mood stabilizer  adjunct medication  anticonvulsant (securing against withdrawal seizures) |
| carbamazepine | mood disorders  personality/behavioral disorder  benzodiazepine dependence | mood stabilizer, adjunct medication  adjunct medication  anticonvulsant, adjunct medication |
| lamotrigine | mood disorders | mood stabilizer |
| trazodone | mood disorders | antidepressant |
| venlafaxine | anxiety disorders  mood disorders | anxiety treatment  antidepressant |
| sertraline | anxiety disorders  mood disorders | anxiety treatment  antidepressant |
| es- or citalopram | anxiety disorders  mood disorders | anxiety treatment  antidepressant |
| tianeptine | mood disorders | antidepressant |
| mianserin | mood disorders | antidepressant |
| agomelatine | mood disorders | antidepressant |
| olanzapine | bipolar mood disorder  depressive mood disorder | antimanic agent  adjunct and sleep-promoting drug |
| quetiapine | bipolar mood disorder  depressive mood disorder | antimanic agent  adjunct medication |
| aripiprazole | bipolar mood disorder | antimanic agent |

Table 1B.

| Medication | Condition |
| --- | --- |
| enalapril, ramipril  telmisartan  amlodipine  bisoprolol | arterial hypertension |
| rova- or atorvastatin | hypercholesterolemia |
| levothyroxine | hypothyroidism |
| doxazosin | prostatic hypertrophy, arterial hypertension |

Supplementary Fig. 1

The S-A-E-R schedule (a model presentation). Reproduced by permission from [28].

The abbreviations: D_E_ – day of completed elimination, D_ACC_ – day of maximum BZD concentration, D_W_ – day of diazepam discontinuation, D_MAX_ and D_LAST_ – days of maximum and last crisis, respectively.

Supplementary Table 2

The most frequent adjunct (accessory) medication is temporarily applied to alleviate BZD-withdrawal symptoms. The drugs were used on demand or temporarily applied on a regular basis.

| Medication | Symptoms |
| --- | --- |
| captopril  atenolol  propranolol  magnesium  ibuprofen  carbamazepine  valproate | elevated arterial pressure  tachycardia, elevated arterial pressure  tachycardia, tremor  tachycardia, tremor, increased muscle tension  headache, muscle pain  neuralgia, paresthesia  anticonvulsant |
| omeprazol  loperamide | gastrointestinal problems |
| hydroxyzine  promethazine  tiaprid  quetiapine | agitation, anxiety, sleep disturbances |
| trazodone  mianserin  mirtazapine  promazin | sleep disturbances |

Supplementary Table 3

For a summary treatment presentation, the measured time values for treatment milestones and some derivatives are presented as median and interquartile (in square brackets) values. Asterisks in the VAL column indicate significant differences between NONE and VAL groups (the CBZ group, as unmatched with respect to initial conditions for the effective elimination process, C_ACC_, has not been included in intergroup comparisons and the CBZ data are presented for informative purposes only). In lower rows, if present, the average values and standard deviations are shown for informative purposes.

| Group | NONE | VAL | CBZ |
| --- | --- | --- | --- |
| the day of concentration culmination, D_ACC_ | 6 [3-8] | 5 [2-7] | 5 [4-7] |
| the duration of the effective elimination,  D_ACC_ to D_E_ | 21 [18-36]  26.8 (16.5) | 29 [22-42]*  32.8 (15.2) | 26 [21-33]  28.2 (12.3) |
| elimination after drug discontinuation,  D_W_ to D_E_ | 18 [10-30]  21.4 (14.5) | 23.5 [15-36]*  26.6 (15.8) | 18 [13-24]  20.8 (12.7) |
| the final day of elimination, D_E_ | 29 [22-45]  33.6 (18.5) | 35 [27-48]  37.5 (16.1) | 31 [25-39]  33.6 (13.0) |
| the day of the last crisis, D_E_ | 30 [21-41]  32.5 (19.2) | 29.5 [19-40]  30.1 (15.6) | 27 [18-40]  30.4 (17.9) |
| the day of the maximal crisis, D_MAX_ | 13.5 [6.5-28]  18.4 (17.2) | 18 [8.5-27.5]  19.1 (12.6) | 17 [11-27]  20.3 (12.6) |
| treatment duration | 51 [35-60] | 58 [38-62] | 50 [33-58] |

Supplementary Table 4

The key study tests for overall population prior (Table 4A, c.f. Table 3) and after (Table 4B) the CBZ group was excluded (a sensitivity check). Abbreviations indicate days counted from the first day of the procedure: D_E_ – day of completed elimination, D_ACC_ – day of maximum BZD concentration, D_W_ – day of diazepam discontinuation, D_MAX_ and D_LAST_ – days of maximum and last crisis, respectively. Likewise in Table 3-6, an additional column for BF% tests has been provided.

The comparison has been done on the data subset used and explained in Supplementary Analysis).

Table 4A

| TOTAL  N = 290 | age  (rho, p) | BMI  (rho, p, (R^2)^) | BF%  (rho, p,( R^2)^) |
| --- | --- | --- | --- |
| the final day of elimination, D_E_ | +0.23, <0.001 | +0.41, <0.001 (0,13) | +0.37, <0.001 (0.15) |
| the duration of the effective elimination,  D_ACC_ to D_E_ | +0.24, <0.001 | +0.38, <0.001 (0.11) | +0.37, <0.001 (0.15) |
| elimination after drug discontinuation,  D_W_ to D_E_ | +0.33, <0.001 | +0.39, <0.001 (0.11) | +0.44, <0.001 (0.18) |
| the day of the strongest crisis, D_MAX_ | +0.13, <0.05 | +0.13, <0.015 (0.01) | +0,15, <0.05 (0.03) |
| the day of the last crisis, D_LAST_ | +0.28, 0.001 | +0.16, =0.01  (0.01) | +0.28, <0.001 (0.07) |
| Notion: the correlation age x BMI is nonsignificant | | |  |

Table 4B

| TOTAL (NONE+VAL)  N = 171 | age | BMI | BF% |
| --- | --- | --- | --- |
| the final day of elimination, D_E_ | +0.21, <0.01 | +0.41, <0.001 (0.12) | +0.33, <0.001 (0.11) |
| the duration of the effective elimination,  D_ACC_ to D_E_ | +0.26, =0.001 | +0.39, <0.001 (0.11) | +0.35, <0.001 (0.13) |
| elimination after drug discontinuation,  D_W_ to D_E_ | +0.37, <0.001 | +0.39, <0.001 (0.11) | +0.40, <0.001 (0.16) |
| the day of the strongest crisis, D_MAX_ | +0.25, <0.01 | +0.10, ns (0.01) | +0.10, ns, (0.01) |
| the day of the last crisis, D_LAST_ | +0.22, <0.01 | +0.14, ns (0.01) | +0.20, <0.01 (0.05) |
| Notion: the correlation age x BMI is nonsignificant (e.g., ρ = 0.08, p= 0.30 here) | | |  |
