# Supplementary material for: Longer Duration of Drug Elimination and Withdrawal Symptoms in Overweight Patients Undergoing Detoxification for Benzodiazepine Dependence
Source: Alpha Psychiatry. 2026 Aug 20;27(4):51895. doi: 10.31083/AP51895 (PMC13540023; doi:10.31083/AP51895)
Supplement: Supplementary file 1 [file 2757-8038-27-4-51895-s1.zip › Supplementary Analysis.docx]

# Supplementary Analysis

# Detailed regression results.

Results of Pearson’s linear regression are shown on corresponding scatterplots. Also, the numeric data from Spearman’s quadratic, HC3 and bootstrap analyses are shown in test boxes. For the whole dataset, the coefficients and CIs are listed. For subgroups (patients medicated by valproate, carbamazepine or none) only the linear regression lines are drawn. The LOESS curve is shown for visual verification of adequacy of linear regression. Quadratic regression results are shown to somewhat quantify possible nonlinear relationships.

As a check on stability of the regression results, both HC3 and bootstrap CIs (95%) are provided. A near agreement of these error estimates is generally interpreted as an argument for stability of the regression approximation. To account for possible heteroskedasticity, a “pessimistic” HC3 p-value is provided, along with standard Pearson’s and Spearman’s p-values.

This supplements the data quoted in the main paper, where mainly the Spearman’s regression was used to argue and quantify the relevance of investigated relationships.

For some technical details (on the dataset used), see the end of this supplement.

## Supplementary Fig. 2 Full dataset.

**Fig. 2A**  The rough agreement of H3C and bootstrap CI estimates, together with reasonable (+-~30%) span of CIs suggests stability, the regression results do not depend meaningfully on sampling.The LOESS fit for DE suggests that at high BMI the “signal from BMI”, if present at all, could be non-linear. As there are only 6% (n=16) of datapoints with BMI > 32, it is pointless to draw conclusions except that it is necessary to gather more data. It is possible that at high BMI some qualitatively different phenomena may be occurring. There is also a possibility that at high BMI, in the population of the patients, BMI might become a proxy for some other than adiposity, "causatively important” variables. In the second part (Supplement 6B) the analysis is repeated for data points with BMI <= 32 showing a good linear behavior.

**Fig. 2B**: a similar situation with stability (good) as in the previous case. Similarly a possible regime change at high BMI, no conclusions possible due to sparse dataset in this region. See Supplement 6B.

**Fig. 2C**: a similar situation to the two previous ones, with a stronger possible nonlinear component. Restricting the dataset (see Supplement 6B) to BMI <=32 (decrease of N by 6%) removes nonlinearity almost completely, improves Pearson’s correlation but worsens Spearman’s correlation and pushes the slope towards the end of CI. All this suggests that the apparent deviation of (DE-DW) from linear behavior for high BMIs might be more than a noise, but of course the data is way too sparse for any conclusion.

**Fig. 2D**: as the slope CI includes 0, we cannot claim a robust correlation here. Still, the CIs are nearly all on the positive side and consistent between HC3 and bootstrap. The regression analysis of DMAX and BF% (not shown here) shows a moderately better correlation, with stronger coefficients and no inclusion of slope=0 in CIs. But “falling back” on another metric (BF% in place of BMI) in mid-study is not a good practice. In future work on adiposity influence, BF% should be strongly considered as an a priori metric, as it removes a part of age and sex “noise”. Alternatively, a multiple regression including BMI, age and sex could be used. As the very notion of “strongest crisis” is not objective, the weak result of regression fitting here is not surprising.

**Fig. 2E**: as in the DMAX case, the result seems very weak. The confidence slopes are (relative to slope) high and include 0, suggesting instability. On the other hand, the correlation (below) between BF% and DLAST looks more clean and stable. This should be investigated further, both the “statistical failure” in this case of the BMI metric as a predictor, and a possible success of BF% in this role (for this particular clinical outcome). Understanding the reason for this could possibly be useful and revealing.

**Fig. 2F:** this scatterplot (correlation with BF%) is provided only as qualitative input for design of a possible further investigation on correlations and possibly influence of adiposity in BZD detoxification treatment. In this particular case, the DLAST variable, BF% provides a better linear correlation than BMI does. Note that we used here not the “real” BF%, but its approximation from the Deurenberg formula.

## Supplementary Fig. 3 Detailed regression results. Dataset limited to BMI <= 32 (N reduced by 6%).

Fig. 3A Removing data points with extreme (>32) BMI did not change the linear part of the correlation meaningfully but the non-linearity remains. The correlation can be treated as robust.

Fig. 3B As with DE, removing data points with extreme (>32) BMI did not change the linear part of the correlation meaningfully but the non-linearity remains. The correlation can be treated as robust. Due to the displayed non-linearity, probably only in the BMI 18-32 range a linear approximation is useful.

Fig. 3C a near perfect case of a “linear influence of a weak signal”. Removing the BMI > 32 datapoints also removed non-linearity. This period of treatment, when drug dosing stopped, was expected to present the simplest picture in statistical analysis. In view of this, the anomalies observed at BMI > 32 (Supplement 6A) raise interest and should be investigated if possible.

Fig. 3D the confidence intervals for correlation slope are about [0,1], with slope = 0.5. We can claim we have a positive robust correlation, but not much beyond this. Still, as the positive correlation seems to reliably exist, this could be a valuable test when constructing physiology-based models of the detoxification treatment. Note that the fact that after discarding datapoints with BMI > 32 we are able to claim some correlation, does not necessarily mean that the BMI > 32 datapoints were pure noise.

Fig. 3E Restricting the dataset to BMI <= 32 shows a modest correlation (R^2^ = 0.031) of DLAST with BMI.

## Technical remarks.

### On the dataset used for scatterplots and sensitivity/robustness analysis

The data was extracted from medical records created over years by different people. Some fields were missing. This causes a slightly different number of valid datapoints for each correlation calculation, but differences are small. After creating scatterplots it became obvious that sometimes personnel were perhaps estimating a patient's BMI instead of calculating it. These entries are forming visible vertical lines in scatterplots. Removing them (for a test) from the dataset did not meaningfully change results, so it was assumed that these likely “hand entries” provide close enough estimated values and are valid.

The requirement to provide a much more elaborate statistical analysis and graphic presentation (this Supplement) came from the reviewers. The extremely short time allowed for paper revision forced the authors to use an outside help to do the statistics and programming work. This involved exporting the study dataset out of the authors’ institution (with obvious issues to be addressed). It turned out that the thus obtained dataset has slightly fewer data points (7% less) than the original collection of non-anonymized data the authors originally worked on. There was no time to investigate the flaw in the formal export procedure, so it was assumed that the dataset analysed here (this Supplement) is a random subsampling of the original dataset. This is not a serious methodological flaw, as the original dataset itself was a sampling of historic records, sampling shaped by issues like legibility of different people's handwriting etc.
As expected (a smaller sample), the correlation numbers and confidence factors obtained in this Supplement are slightly worse than what the authors put in the first version of the paper, but differences are small. One can treat this as a yet another test for robustness of the original conclusions. The results in this Supplement come from this smaller (by about 7%) dataset.

### On the technical aspects of extending the adiposity metrics set

The idea of using other than BMI adiposity metrics came only during the paper review process. After some statistical work it became clear that a separate paper should be prepared to properly present the topic. For example, the analysis design and the workflow for investigating BMI dependencies should be different from the workflow using BF%, as the latter (or, more precisely, the method used to **compute** the estimate of BF% from the available data) depends on more input variables, in addition to BMI. This necessitates additional analytic work for BF% (like accounting for possible internal dependencies in the dataset, population issues etc). Because of the above, the different metrics are only briefly mentioned in this paper.

### On the tools used for scatterplots and sensitivity/robustness analysis

The presented results were calculated using a custom Python script and statsmodel and scipy libraries. We used 1000 bootstrap iterations in each analysis case. This pertains only to this Supplement. The analysis presented in the main paper was prepared using the Statistica software version 13.3. See the “On the dataset…” subsection above.
